# Serum Antibody Response Comparison and Adverse Reaction Analysis in Healthcare Workers Vaccinated with the BNT162b2 or ChAdOx1 COVID-19 Vaccine

**DOI:** 10.3390/vaccines9121379

**Published:** 2021-11-24

**Authors:** Sung-Hee Lim, Seong-Hyeok Choi, Bora Kim, Ji-Youn Kim, Young-Sok Ji, Se-Hyung Kim, Chan-Kyu Kim, Tark Kim, Eun-Ju Choo, Ji-Eun Moon, Jina Yun, Seong-Kyu Park

**Affiliations:** 1 Department of Medicine, Division of Hematology-Oncology, Soonchunhyang University Bucheon Hospital, Bucheon 14584, Korea; polluxis82@gmail.com (S.-H.L.); 20210225@schmc.ac.kr (S.-H.C.); 20161294@schmc.ac.kr (B.K.); banji79@schmc.ac.kr (J.-Y.K.); 106258@schmc.ac.kr (Y.-S.J.); shkim@schmc.ac.kr (S.-H.K.); md53097@schmc.ac.kr (C.-K.K.); 2 Department of Medicine, Division of Infectious Disease, Soonchunhyang University Bucheon Hospital, Bucheon 14584, Korea; ktocc@schmc.ac.kr (T.K.); mdchoo@schmc.ac.kr (E.-J.C.); 3 Department of Biostatistics, Soonchunhyang University Bucheon Hospital, Bucheon 14584, Korea; moon6188@schmc.ac.kr

**Keywords:** ChAdOx1, BNT162b2, SARS-CoV-2 vaccines, neutralizing antibodies immunogenicity

## Abstract

The COVID-19 pandemic is changing rapidly and requires different strategies to maintain immunization. In Korea, different COVID-19 vaccines are recommended and available for various populations, including healthcare workers (HCWs) at high risk of SARS-CoV-2 infection. We plan to evaluate the adverse events (AEs) and immunogenicity of the BNT162b2 and ChAdOx1 vaccines in HCWs at a single center. This cohort study included HCWs fully vaccinated with either BNT162b2 or ChAdOx1. Blood samples were taken eight weeks after the second vaccination with both COVID-19 vaccines and six months after the second vaccination from participants with the BNT162b2 vaccine. The primary endpoint for immunogenicity was the serum neutralizing antibody responses eight weeks after vaccination. The secondary endpoint was the incidence of various AEs within 28 days of each vaccination. Between 16 March and 23 June 2021, 115 participants were enrolled (65 in the ChAdOx1 group and 50 in the BNT162b2 group). Significantly higher surrogate virus neutralization test (sVNT) inhibition was observed in participants vaccinated with two doses of BNT162b2 (mean (SD) 91.4 (9.68)%) than in those vaccinated with ChAdOx1 (mean (SD) 73.3 (22.57)%). The effectiveness of the BNT162b2 vaccine was maintained across all age and gender categories. At six months after the second dose, serum antibody levels declined significantly in the BNT162b2 group. The main adverse events, including fever, myalgia, fatigue, and headache, were significantly higher in the ChAdOx1 group after the first dose, whereas, after the second dose, those AEs were significantly higher in the BNT162b2 group (*p* < 0.05). Two doses of either the ChAdOx1 or the BNT162b2 COVID-19 vaccine resulted in very high seropositivity among the HCWs at our center. The quality of the antibody response, measured by sVNT inhibition, was significantly better with the BNT162b2 vaccine than with the ChAdOx1 vaccine. There was no significant association between neutralizing antibody response and AE after each vaccination in our cohort.

## 1. Introduction

Coronavirus disease 2019 (COVID-19) is a respiratory tract infection caused by the severe acute respiratory syndrome coronavirus (SARS-CoV-2), which initially emerged in China in late 2019 [1]. The rapid global spread of this novel virus led the WHO to declare a pandemic in March 2020. As of July 2021, there have been more than 180 million confirmed cases with 3.93 million deaths worldwide [2], and 157,723 confirmed cases with 2021 deaths reported in Korea [3].

Several vaccines against COVID-19 have been approved globally, and some are currently ongoing in human trials. There are two kinds of COVID-19 vaccines available at our center. One is a messenger RNA (mRNA)-based vaccine (BNT162b2) developed by Pfizer/BioNTech. This lipid nanoparticle-formulated, nucleoside-modified RNA vaccine encodes a prefusion-stabilized, membrane-anchored SARS-CoV-2 full-length spike protein [4]. The other is a genetically modified organism (virus vector) vaccine (ChAdOx1), developed by both the University of Oxford and AstraZeneca, which consists of the replication-deficient chimpanzee adenoviral vector ChAdOx1, containing the SARS-CoV-2 structural surface glycoprotein antigen (spike protein; nCoV-19) gene [5]. However, the landmark mRNA COVID-19 vaccine phase 3 trials included less than 5% Asian participants [4,6]. ChAdOx1 has only been tested for western countries.

Several outbreaks of COVID-19 have emerged in hospitals, and healthcare workers (HCWs), as well as patients, are at a high risk of contracting COVID-19 infection. In this study, we aimed to compare the neutralizing antibodies with a surrogate virus neutralization test, and analyze the adverse events of two different COVID-19 vaccines in HCWs at a single-center, real-world setting in Korea. 

## 2. Methods

### 2.1. Study Design and Participants

This study was conducted as a cross-sectional study. From 16 to 18 March 2021 and 6 to 8 April 2021, a total of 2271 HCWs at the Soon Chun Hyang University Bucheon Hospital were vaccinated with two doses of BNT162b2 (Pfizer) three weeks apart. From 29 to 31 March and 22 to 23 June, 303 HCWs received two doses of ChAdOx1 vaccine (AstraZeneca/Oxford) 12 weeks apart. Among them, we enrolled HCWs who were interested and participated voluntarily in this study. Healthcare workers who had ever had COVID-19 were excluded. Participants who consented to the study were given a self-administered questionnaire for adverse events of vaccination. The questionnaire included demographics (age, gender), type of vaccination, previous history of confirmed SARS-CoV-2 infection, pre- and post-vaccination medications, and need for medical attention. All participants had no history of COVID-19 infection or suspected symptoms at the time of registration. 

We collected blood samples from participants eight weeks after the second dose. Because of the different vaccination schedules, we decided to collect blood samples from the ChAdOx1 vaccine group after the first dose. Blood samples were also taken from 10 HCWs who were not vaccinated as controls. All blood samples were analyzed using the commercial virus neutralization test kit (Genscript Biotech Corporation, Piscataway, NJ, USA). In the BNT162b2 group, another blood sample was drawn six months after the second dose. 

The acquired data included the following: sex, age, date of vaccination, history of COVID-19/drug AEs/allergy, types and duration of adverse events, use of drugs (acetaminophen, ibuprofen, opioid) and visits to an outpatient clinic or emergency room. The institutional review board (IRB) of Soon Chun Hyang University Bucheon Hospital approved this study (IRB No. 2021-07-027). Written consent was obtained from all enrolled participants. 

### 2.2. Serological Assays

We used the surrogate virus neutralization test for both SARS-CoV-2 vaccines. Circulating neutralizing antibodies (Nabs) were detected using the GenScript SARS-CoV-2 surrogate virus neutralization test (sVNT) kit (Genscript Biotech Corporation), with excellent correlation of the conventional VNT and pseudovirus-based VNT [7]. The surrogate virus neutralization test (sVNT) is based on blocking ELISA methodology, mimicking virus–cell interactions to detect the presence of neutralizing antibodies in a blood sample. ACE2 protein is plated and HRP-labeled RBD (receptor binding domain) (HRP-RBD) is used for detection.

The sVNT kit results were interpreted by the inhibition rate, which was calculated as follows:

$$\mathrm{Inhibition}\;=\;(1\;-\;\frac{\mathrm{OD}\;\mathrm{value}\;\mathrm{of}\;\mathrm{sample}}{\mathrm{OD}\;\mathrm{value}\;\mathrm{of}\;\mathrm{negative}\;\mathrm{control}})\;\times \;100\%$$

It was classified into positive and negative samples with a 30% cutoff [8]. Samples with more neutralizing antibodies show lower signal intensity, thus sVNT inhibition (%) score represents the number of presenting neutralizing antibodies of SARS-CoV-2 [9].

The US FDA recommends detected neutralizing antibody titers in COVID-19 convalescent plasma of at least 1:160. A qualifying result by the Genscript SARS-CoV-2 neutralization antibody detection kit is an inhibition percent more than 68 [10]. 

### 2.3. Outcomes

The primary objective was to evaluate the immunogenicity of the two different COVID-19 vaccines and the secondary endpoint was to evaluate their safety. 

### 2.4. Statistical Analysis

All measurements and calculation data were presented as the mean ± standard deviation (SD), median, IQR, range for continuous variables and frequency (percentage) for categorical variables. To compare the variables between independent groups, either independent two sample *t*-tests or chi-square tests (also known as Fisher’s exact tests) were used where appropriate. To compare the variables within one subject, a paired *t*-test was used. Multivariable liner regression analysis was performed using a stepwise method that included all variables (age, sex, type of vaccine, AE to the first dose, AE to the second dose, rescue medication) to find the predictors of sVNT inhibition (%). The collinearity between variables was verified. The regression coefficients (β) and their 95% confidence intervals (CI) were also calculated. All statistical analyses were performed using IBM SPSS Statistics version 27 (IBM Corp., Armonk, NY, USA) and GraphPad Prism version 9.0 (GraphPad Software).

## 3. Results

This study included a total of 115 HCWs, with 65 participants in the ChAdOx1 group and 50 participants in the BNT162b2 group.

### 3.1. Demographics of Participants

The mean age of all 115 participants was 42.5, ranging from 23 to 72 years old. The ChAdOx1 vaccine group comprised 16 men (25%) and 49 women (75%) (mean (SD) age of 41.7 (13.60) years), and the BNT162b2 vaccine group comprised 11 men (22%) and 39 women (78%) (mean (SD) age of 43.5 (9.35) years) (Table 1). Most participants had no history of any allergy or anaphylaxis to drugs or foods. One participant in the ChAdOx1 group had previous vaccine anaphylaxis. The mean/median time between the second vaccine dose and the blood sample draw was 56.9/56 days (54–63) in the BNT162b2 group and 56.1/56 (41–57) days in the ChAdOx1 group (*p* = 0.999).

### 3.2. Serology Results

**Figure 1:**
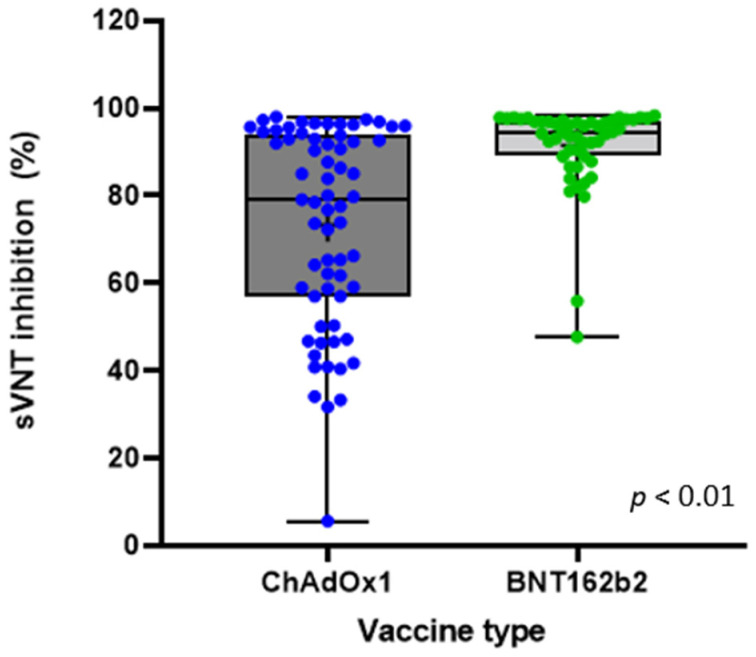
Neutralizing antibody level measured by percentage inhibition of sVNT readings in both vaccine groups.

Significantly higher sVNT inhibition was observed in the participants vaccinated with two doses of BNT162b2 (mean (SD) 91.4 (9.68)%, median 94.5%, Q1/Q3 89.4/97.1) compared with those vaccinated with ChAdOx1 (mean (SD) 73.3 (22.57)%, median 79.1%, Q1/Q3 57.0/93.8) (*p* < 0.01) (Figure 1, Table 2). In the ChAdOx1 group, 64 (98.5%) had a positive serologic test result, and in the BNT162b2 group, 100% had a positive serologic test result. In the ChAdOx1 group, 60% (*n* = 39) had positive results, with over 68% sVNT inhibition, compared with 96% (*n* = 48) in the BNT162b2 group, which was a significant difference (*p* < 0.01) (Table 2). Among the 10 non-vaccinated control HCWs, one was seropositive (10%) and the mean (SD) of sVNT inhibition was 13.31 (12.09)%. The one seropositive, non-vaccinated HCW had no history of COVID-19 infection. 

According to gender, a significantly higher mean sVNT score was observed in the participants vaccinated with two doses of BNT162b2 than in those vaccinated with ChAdOx1 (Supplementary Figure S1). In both the vaccine groups, the sVNT scores of female participants were slightly higher compared with male participants, without significance (mean (SD) 87.46 (14.46)% vs. 92.55 (7.74)%, respectively, in the BNT162b2 group, *p* = 0.125; 70.28 (23.98)% vs. 74.22 (22.26)%, respectively, in the ChAdOx1 group, *p* = 0.567). Across all the age categories, the participants vaccinated with BNT162b2 had higher sVNT scores than those vaccinated with ChAdOx1 (*p* < 0.05 for all comparisons; Supplementary Figure S1).

**Figure 2:**
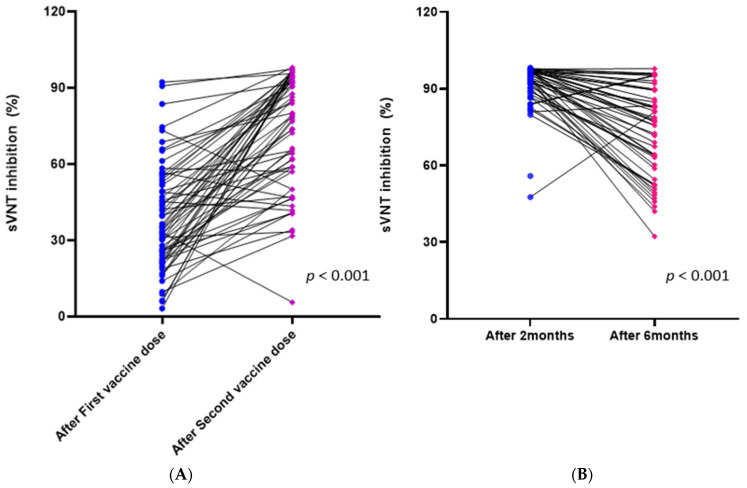
(**A**) Changes in sVNT inhibition (%) scores after the first and second doses in the ChAdOx1 group, (**B**) changes in sVNT inhibition (%) scores at two months and six months after the second dose in the BNT162b2 group.

Among the 65 participants vaccinated with ChAdOx1, the sVNT inhibition (%) scores significantly increased after the second dose compared to after the first dose (*p* < 0.001, mean from 38.25 to 73.25) (Figure 2A). There was no significant predictive factor affecting the difference between the two vaccine doses.

The sVNT inhibition scores negatively correlated with age among all the populations, regardless of the type of vaccine (correlation coefficient, of −0.22, *p* < 0.044). The type of vaccine remained independently associated with higher sVNT inhibition scores in a multiple linear regression (*p* < 0.001) (Supplementary Table S1).

### 3.3. Changes in Immune Response in the BNT162b2 Group

Among the 50 HCWs fully vaccinated with the BNT162b2 vaccine, 49 blood samples were drawn six months after the second vaccination. The sVNT inhibition (%) scores significantly decreased after six months, compared to the first sampling two months after the second vaccination (*p* < 0.001, mean from 92.16 to 72.29) (Figure 2B). The mean (SD) of the difference was 19.87 (18.56), 14.3% of the cases had a difference greater than 40, and five participants (10.2%) had increased sVNT inhibition (%) scores at six months. Although there were no significant factors associated with the degree of decreased antibody response, a trend showing a positive correlation of the degree of decrease in sVNT with increasing age was observed (correlation coefficient of 0.519 (95% CI: −0.54–1.092), *p* = 0.075). 

### 3.4. Adverse Events

**Figure 3:**
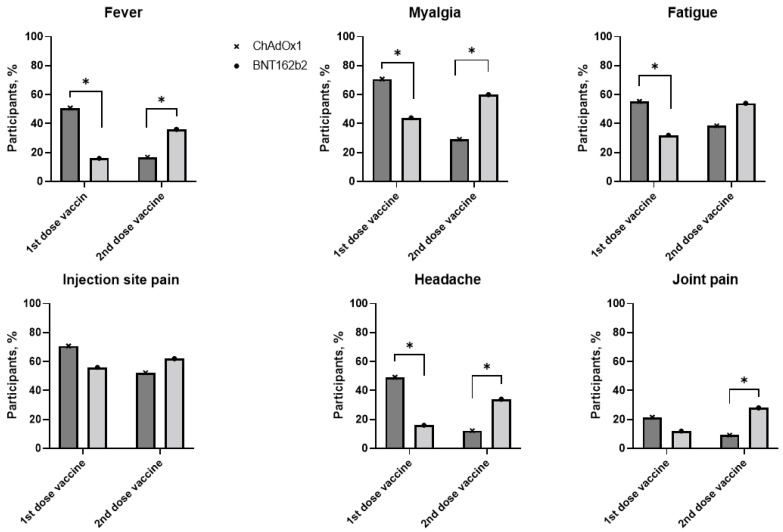
Most commonly reported adverse events after the first and second doses in ChAdOx1 and BNT162b2 groups. * *p* value < 0.05 when comparing frequencies of adverse events between ChAdOx1 and BNT162b2 groups.

Systemic or local side effects of the first dose were reported in 92% (*n* = 60) of participants in the ChAdOx1 group and 80% (*n* = 40) in the BNT162b2 group. However, after the second dose, more side effects were reported in the BNT162b2 group, up to 92%, compared with 80% in the ChAdOx1 group. After the first dose, the participants vaccinated with ChAdOx1 had significantly higher incidence of fever, myalgia and headache AEs compared with those vaccinated with BNT162b2, and incidences of these AEs were significantly lower in the ChAdOx1 group after the second dose (Figure 3). The most commonly reported systemic reactions in both the vaccine groups were myalgia, fatigue, fever, chills, and headache. Pain at the injection site was the most commonly reported local reaction, around 60%, in both the vaccine groups. Detailed systemic and local adverse reactions within 28 days after the first and second doses are listed in Supplementary Table S2.

## 4. Discussion

This cohort study demonstrated a significantly higher humoral immunogenicity of the SARS-CoV-2 BNT162b2 mRNA vaccine compared with the ChAdOx1 vector vaccine in healthy healthcare workers. Using a surrogate virus neutralization test (sVNT), we found that 64 (98.5%) of the 65 participants vaccinated with ChAdOx1, and 100% of the participants vaccinated with BNT162b2, were seropositive for SARS-CoV-2 Nab eight weeks after receiving the second dose. However, the sVNT inhibition score was significantly higher in the BNT162b2 group than in the ChAdOx1 group (means of 91.44 vs. 73.25%, respectively; *p* < 0.01).

Neutralization assays are the gold standard for determining if a patient has effective antibodies and protective immunity against SARS-CoV-2 [11]. Several studies reported that the neutralization level is highly predictive of immune protection [12,13]. The levels of neutralizing antibodies, as determined by sVNT, are strongly correlated with anti-SARS-CoV-2 S1 IgG and IgA antibodies [14]. They were also positively correlated with the duration and severity of the clinical symptoms of convalescent COVID-19 patients [14]. 

Although both vaccines showed almost 100% seropositivity, we found that the BNT162b2 vaccine showed significantly higher protective immune responses (96%) than the ChAdOx1 vaccine (60%), based on the cut off of over 68% sVNT inhibition (*p* < 0.01). This result was similar to previously published phase 3 clinical trials. A phase 3 trial of the Pfizer–BioNTech BNT162b2 messenger RNA (mRNA) vaccine demonstrated 95% efficacy in preventing SARS-CoV-2 infection seven days after the second dose [4], and a pivotal trial of the ChAdOx1 vaccine showed that the overall vaccine efficacy after two standard doses was 62.1% [5]. This suggests that sVNT may be useful for evaluating humoral immunogenicity after SARS-CoV-2 vaccination, as well as SARS-CoV-2 infection. 

Recently, another group suggested that there is an association between antibody response and reactogenicity after anti-SARS-CoV-2 vaccination [15]. In this study, the sVNT inhibition scores positively correlated with experiencing more than three AEs after the second dose (correlation coefficient of 12.76, *p* <0.025) in a univariate analysis; however, in a multivariate analysis, the correlation between the number of AEs and sVNT inhibition scores was not significant. Interestingly, the incidence of the main side effects of the BNT162b2 vaccine was significantly higher compared to that with the ChAdOx1 vaccine after the second dose. After the first dose of the ChAdOx1 vaccine, significantly higher AEs were reported compared to the BNT162b2 vaccine, similar to a previous study [16].

This study has limitations. The sample size was relatively small for each vaccine group. The median age of all the participants was 42.5 years and more than 75% were female. Therefore, the results of this study might not be generalizable to other populations, who might have different immune profiles. Lastly, circulating neutralizing antibodies might not be enough to reflect a robust immune response. There is an opinion that SARS-CoV-2-specific memory T cells and B cells are important for long-term protection [17]. 

Recent reports have shown that antibody levels significantly decline at six months after the second dose of the BNT162b2 mRNA vaccine [18,19]. Our study also showed a significant decrease in sVNT inhibition scores at six months, compared to two months after completion of the second vaccination. We also plan to assess the changes in antibody levels after six months of vaccination in the ChAdOx1 cohort. Our study shows quite different antibody responses and side effects of the two COVID-19 vaccines in a real-life vaccination situation. As our results show differences in the neutralizing antibody value (sVNT) and changes in antibody response over time between the two vaccines, different vaccines and tailored vaccine schedules offer an opportunity to further optimize vaccine response. 

Further research should address the dynamics of the antibody responses of different COVID-19 vaccines for a longer term, and recommendation for individualized vaccination is warranted.

## Figures and Tables

**Table 1:** Demographics of participants.

Variables	ChAdOx1 (AstraZeneca/Oxford) Group (*n* = 65)	BNT162b2 (Pfizer) Group (*n* = 50)	*p* Value
Age, years			
Mean ± SD (range)	41.7 ± 13.6 (23–72)	43.5 ± 9.35 (24–60)	0.411
Sex			0.826
Male	16 (24.6)	11 (22.0)	
Female	49 (75.4)	39 (78.0)	
Previous allergy/anaphylaxis history			0.079
None	62 (95.4%)	41 (82.0%)	
Drug/Food	2 (3.1%)	5 (10.0%)	
Anaphylaxis to drug/food	0	2 (4.0%)	
Vaccine allergy	0	2 (4.0%)	
Vaccine anaphylaxis	1 (1.5%)	0	
Days post 2nd vaccination, mean/median (range)	56.1/56.0 (41–57)	56.9/56.0 (54–63)	0.999

**Table 2:** Serology results after 2nd dose of vaccine.

	ChAdOx1 (AstraZeneca/Oxford) Group (*n* = 65)	BNT162b2 (Pfizer) Group (*n* = 50)	
Seropositive, No (%)	64 (98.5)	50 (100)	0.999
sVNT inhibition (%)			
Mean ± Std	73.25 ± 22.57	91.44 ± 9.68	<0.01
Median	79.05	94.48	
Q1, Q3	57.01, 93.76	89.37, 97.08	
Range	5.64~97.94	47.67~98.25	
Presumed Protection Rates of COVID-19 *			
≥68% *, No (%)	39 (60.0)	48 (96.0)	<0.01
<68%, No (%)	26 (40.0)	2 (4.0)	

sVNT, surrogate virus neutralization test; Std, standard deviation. * sVNT inhibition score ≥ 68% is suggestive of enough protection against COVID-19 infection.

## Data Availability

The data presented in this study are available on request from the corresponding author. The data are not publicly available due to restricted consent.

## Supplementary Materials

The following are available online at https://www.mdpi.com/article/10.3390/vaccines9121379/s1, Supplementary Figure S1: Violin plots of neutralizing antibody level measured by percentage inhibition of sVNT readings in both groups, Table S1: Multivariable linear regression model of sVNT inhibition noted antibody levels after 2nd dose of two kinds of COVID-19 vaccine, Table S2: Adverse reactions within 28days after First and Second vaccine dose.
