# Supplementary material for: Serum Antibody Response Comparison and Adverse Reaction Analysis in Healthcare Workers Vaccinated with the BNT162b2 or ChAdOx1 COVID-19 Vaccine
Source: Vaccines (Basel). 2021 Nov 24;9(12):1379. doi: 10.3390/vaccines9121379 (PMC8708890; doi:10.3390/vaccines9121379)

Supplementary Table S1. Multivariable linear regression model of sVNT inhibition noted antibody levels after 2nd dose of two kinds of COVID-19 vaccine

	Multivariable analysis	
	**β (95% CI)**	**P value**
**Age**	-0.29 (-0.56-0.008)	0.044
**Sex**		
**Male**	[Reference]	
**Female**	5.06 (-3.73-13.86)	0.256
**Vaccine type**		
**ChAdOx1**	[Reference]	
**BNT162b2**	18.69 (12.00-25.38)	<0.001

Abbreviations: NA, not applicable; CI, confidence interval

Supplementary Table S2. Adverse reactions within 28days after First and Second vaccine dose

Adverse reactions	1st dose vaccine			2nd dose vaccine		
	BNT162b2 group (N=50)	ChAdOx1 group (N=65)	p-value	BNT162b2 group (N=50)	ChAdOx1 group (N=65)	p-value
Any	40 (80.0%)	60 (92.3%)	0.091	46 (92%)	52 (80%)	0.111
Systemic						
Fever	8 (16.0%)	33 (50.8%)		18 (36.0%)	11 (16.9%)	
Chills	8 (16.0%)	37 (56.9%)		15 (30.0%)	3 (4.6%)	
Myalgia	22 (44.0%)	46 (70.8%)		30 (60.0%)	19 (29.2%)	
Headache	8 (16.0%)	32 (49.2%)		17 (34.0%)	8 (12.3%)	
Nausea	3 (6.0%)	8 (12.3%)		5 (10.0%)	1 (1.5%)	
Vomiting	2 (4.0%)	3 (4.6%)		2 (4.0%)	-	
Fatigue	16 (32.0%)	36 (55.4%)		27 (54.0%)	25 (38.4%)	
Joint pain	6 (12.0%)	14 (21.5%)		14 (28.0%)	6 (9.2%)	
Dyspnea	1 (2.0%)	3 (4.6%)		1 (2.0%)	-	
Dizziness	4 (8.0%)	5 (7.7%)		5 (10.0%)	2 (3.1%)	
Rash	-	-		-	-	
Pruritus	1 (2.0%)	3 (4.6%)		1 (2.0%)	1 (1.5%)	
Anaphylaxis*	-	-		-	-	
Injection site						
Pain	28 (56.0%)	46 (70.8%)		31 (62.0%)	34 (52.3%)	
Heating sense or Redness	7 (14.0%)	11 (16.9%)		4 (8.0%)	5 (7.7%)	
Swelling	8 (16.0%)	9 (13.8%)		8 (16.0%)	4 (6.2%)	
Timing of AE after vaccination						
Within 3hours	6 (12.0%)	3 (4.6%)		4 (8.0%)	8 (12.3%)	
3-6 hours	12 (24.0%)	11 (16.9%)		15 (30.0%)	13 (20%)	
6-12 hours	12 (24.0%)	32 (49.2%)		14 (28.0%)	15 (23.1%)	
12-24 hours	5 (10.0%)	9 (13.8%)		11 (22.0%)	7 (10.8%)	
24-48 hours	4 (8.0%)	3 (4.6%)		2 (4.0%)	5 (7.7%)	
After 48 hours	1 (2.0%)	1 (1.5%)		-	1 (1.5%)	
Duration of AE						
< 24 hours	10 (20.0%)	8 (12.3%)		8 (16.0%)	25 (38.5%)	
24-48 hours	16 (32.0%)	32 (49.2%)		24 (48.0%)	21 (32.3%)	
48-72 hours	8 (16.0%)	13 (20.0%)		8 (16.0%)	2 (3.1%)	
72 hours – 5 days	3 (6.0%)	4 (6.2%)		3 (6.0%)	-	

More than 5days	2 (4.0%)	2 (3.1%)	3 (6.0%)	2 (3.1%)
Use of medication**				
None	24 (48.0%)	14 (21.5%)	9 (18.0%)	40 (61.5%)
Acetaminophen	25 (50.0%)	49 (75.4%)	40 (80.0%)	24 (36.9%)
Ibuprofen	3 (6.0%)	4 (6.2%)	1 (2.0%)	1 (1.5%)
Anti-histamine	1 (2.0%)	-	1 (2.0%)	-
Steroid	1 (2.0%)	-	1 (2.0%)	-
Visit to hospital				
Outpatient clinic	2 (4.0%)	1 (1.5%)	-	1* (1.5%)
Emergency room	4 (8.0%)	1 (1.5%)	1 (2.0%)	1* (1.5%)
Admission	1 (2.0%)	1 (1.5%)	-	1* (1.5%)

*anaphylaxis is diagnosed by a doctor; **allow double dosing

Supplementary Figure S1. Violin plots of neutralizing antibody level measured by percentage inhibition of sVNT readings in both groups

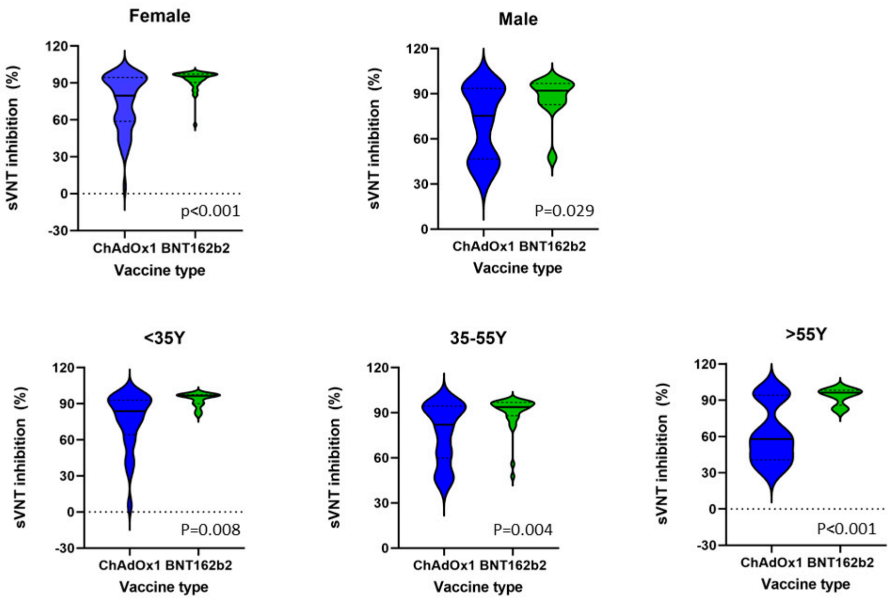

Supplement: Supplementary file 1 [file vaccines-09-01379-s001.zip › vaccines-1470176-supplementary.pdf]
